# Community and health workers’ perspective on impacts of climate change on reproductive, maternal, and child health outcomes in Kilwa district council, Tanzania: a qualitative study

**DOI:** 10.1186/s12889-025-24343-2

**Published:** 2025-09-30

**Authors:** Reebok Mnyigumba, Hussein Mohamed, Sixbert Mwanga, Wande Rajabu, Stelyus L. Mkoma, Boniventure Mchomvu, Sharon Kishenyi, Elifadhili Shaidi, Mkombozi Joaness

**Affiliations:** 1 Department of Research, Innovation and Consulting, Climate Action Network Tanzania, P.O. Box 32900, Dar Es Salaam, Tanzania; 2 Department of Environmental and Occupational Health, School of Public Health and Social Sciences, Muhimbili University of Health and Allied Sciences, P.O. Box 65015, Dar Es Salaam, Tanzania

**Keywords:** Climate change, Extreme Weather Events, Maternal, Reproductive, And Child health

## Abstract

**Background:**

Climate change continues to manifest at an unprecedented rate and is increasingly recognized as a serious threat to reproductive, maternal, and child health. This qualitative study aims to explore community and healthcare workers’ perspectives on the impacts of climate change and related extreme weather events on reproductive, maternal, and child health outcomes in Kilwa District, Tanzania.

**Methods:**

In October 2024, a cross-sectional qualitative study design was employed where 10 focus group discussions with women and 15 key informant interviews with healthcare workers in 10 flood-prone villages in Kilwa District were conducted. All discussions and interviews were audio-recorded, transcribed verbatim, and analyzed using NVivo-12, both inductively and deductively.

**Results:**

Most respondents perceive climate change as a shift in weather patterns over time, as evidenced by rising temperatures, unpredictable rainfall, and an increasing frequency of floods and tropical cyclones. High temperature, floods, and prolonged dry spells were perceived by community and healthcare workers to have direct and indirect impacts on reproductive, maternal, and child health outcomes. Study participants perceived a clear linkage between observed weather changes and negative maternal and child health outcomes, including limited accessibility to health facilities during the flooding season, a surge in climate-sensitive diseases such as malaria and diarrhoea in the aftermath of floods, and increased food insecurity leading to malnutrition. The increased frequency of climate-related disasters has driven changes in fertility intentions, as women have expressed a desire to have smaller families, fearing that climate-related extreme weather events would further strain their financial capacity to support larger families. Participants described that women faced heightened vulnerabilities due to patriarchal gender norms. Travelling long distances searching for water, increased burden of household chores, and climate-induced economic instability were reported to put women and girls at increased risk of experiencing sexual and gender-based violence. In response to these impacts, the community has implemented several adaptation measures such as utilizing maternity waiting homes during the flooding season, provision of psychosocial support for disaster victims, and relocation of vulnerable populations from flood-prone areas.

**Conclusion:**

Community and healthcare workers' perspectives from this study highlight that the ongoing climate crisis is taking a heavy toll on reproductive, maternal, and children's health in Kilwa district. The findings underscore the critical urgency for strengthening the climate resilience of the healthcare system to ensure the continuity of healthcare service provision during and after climate-related disasters. Additionally, policy solutions are proposed to take into account the differential vulnerability and impacts of climate change, thereby ensuring climate action is gender-responsive.

**Supplementary Information:**

The online version contains supplementary material available at 10.1186/s12889-025-24343-2.

## Background

Climate change has emerged as the greatest public health threat of the twenty-first century [17, 26, 29]. Rising global temperatures, changing precipitation patterns, sea level rise, and increasing frequency and intensity of extreme weather events such as floods, heatwaves, droughts, and tropical cyclones have far-reaching impacts on human health, economies, livelihoods, and ecosystems. For the past two decades (2000–2019), a total of 7348 major climate-related disasters occurred worldwide, with Asia recording more disaster events than any other continent in the world (UNDRR, 2021). In 2019 alone, 396 disasters were reported to claim the lives of 11,755 people, affecting more than 95 million, and causing economic loss of nearly $130 billion [18]. Several human activities, such as the use of fossil fuels, deforestation, and agriculture, to mention a few, contribute to increased levels of heat-trapping gases (greenhouse gases) such as carbon dioxide, which is responsible for driving global warming.

The United Republic of Tanzania is among the most climate-vulnerable countries in the world, ranking 40th in vulnerability and 145th in climate change readiness out of 187 countries globally [28]. Several reports indicate Tanzania experiences worsening impacts of climate change, marked by sea level rise, temperature rises, and an increase in frequency and intensity of EWEs such as tropical cyclones, recurring floods, and droughts [25, 37]. Recent meteorological data from the Tanzania Meteorological Authority (TMA) indicate worsening trends. A record-breaking annual average temperature of 24.3 °C and total rainfall of 1307.6 mm were recorded in 2024, making it the warmest year and first wettest in the past two decades, respectively [35]. Exposure to rising temperatures, changing rainfall patterns, and extreme events (e.g., droughts, floods, and high/extreme temperature events) results in a wide range of health-related impacts, including epidemics of climate-sensitive diseases such as malaria, diarrhoea, dengue, cholera, and heat-related illnesses [25, 36]. These climate-related extreme weather events disrupt health systems, damage infrastructure, and limit access to essential services. In addition, surging cases of climate-sensitive diseases and climate-related emergencies put an additional burden on the already fragile healthcare system [25]

Beyond the increased burden of climate-sensitive diseases, recent epidemiological and physiological studies globally indicate that the impacts of changing weather conditions resulting from climate change have direct and indirect impacts on maternal, reproductive, and child health outcomes. Extreme temperature is directly associated with increased risks of adverse birth outcomes such as stillbirth and pre-term birth [8, 23, 34]. Also, higher temperatures have been found to increase the morbidity and mortality rates related to vector-borne diseases and water-borne diseases such as malaria, dengue, and diarrhea among pregnant women and newborn children [3, 5, 14, 22]. Furthermore, studies highlight that malaria during pregnancy can have serious implications for both mother and unborn baby, as it may result in maternal and fatal anemia, miscarriage, preterm birth, low birth weight infants, and maternal and/or neonatal mortality [11, 13, 15, 20, 33].

Moreover, climate-related extreme weather events (EWEs), such as drought and floods, not only trigger outbreaks of waterborne diseases but also interrupt maternal and reproductive health services, reduce antenatal care attendance, increase home deliveries, and heighten maternal and neonatal risks [10, 24, 27, 31, 32]. For example, a study in Bangladesh found that maternal deaths are most likely to occur during the rainy season, particularly in flood-affected areas. Such an increase is due to several factors, such as the unavailability of proper maternal healthcare services, reliance on unqualified traditional birth attendants, communication and transportation challenges, and barriers to timely referral for pregnant women experiencing complications during floods (A. S. M. [1]). Moreover, recurrent droughts and floods reduce agricultural productivity, destabilize household economic status, increase food prices, and drive households'food insecurity. This results in reduced availability of nutritious foods required by pregnant and lactating mothers, resulting in maternal and child malnutrition, which heightens the risks of anemia, low birth weight, and stunting [30].

Despite a dearth of global and regional evidence indicating that climate change and related EWEs can adversely impact reproductive, maternal, and child health outcomes, empirical data specific to the United Republic of Tanzania remains limited. Existing studies largely focus on studying the general health impacts, specifically on climate-sensitive diseases, and not on reproductive and maternal health outcomes. To address this research gap, our qualitative study in Kilwa District Council, Lindi region, explores the community and healthcare providers'perspectives on the impacts of climate change and related extreme weather events on maternal, reproductive, and child health outcomes. The findings generated through this study provide crucial insights that can be used to inform national policies and existing reproductive, maternal, neonatal, Child, and adolescent health programmes and interventions.

## Methods

### Study area

The study was conducted in Kilwa District Council, which is in the Southeast of Tanzania's mainland. It is one among the six councils forming the Lindi Region and covers a total area of 15,000 km^2^. It is bordered to the east by the Indian Ocean and to the west by Nyerere National Park. Kilwa has a coastal climate with an average annual temperature ranging from 22 °C to 30 °C, with 98–100% humidity and a mean annual rainfall of 1034 mm [21]. According to the most recent national population and housing census of 2022, the district has a total population of 297,676 [38]. This district was purposively selected for this study because it is one of the most climate-affected areas in the southern zone of Tanzania, as it has been experiencing multiple climate-related challenges such as recurring floods, prolonged dry spells, and tropical cyclones over time. This exposure to climate-related hazards makes the district an appropriate setting for gathering experience-based insights from community members and healthcare providers on the interconnection between climate change and reproductive, maternal. and child health in the district. Notably, between March and May 2024, the district was also heavily hit by devastating floods and tropical cyclone Hidaya, which left a trail of destruction in farmlands and other critical infrastructures such as roads, bridges, houses, electric poles, schools, and healthcare facilities.

### Study design

This study was a cross-sectional qualitative study design that was purposively employed to gather information from members of the Council Health Management Team, healthcare providers from primary healthcare facilities (gatekeepers in Tanzania’s healthcare system), and women, some of whom were mothers aged 18 years and above. These study respondents were carefully selected to ensure in-depth gathering of lived and observed experiences of the effects of climate change on reproductive, maternal, and child health in Kilwa District Council. The Focus Group Discussions (FGDs) approach was employed to explore understanding of climate variability and its related impacts on reproductive, maternal, and child health outcomes among women who are pregnant, or have given birth in the previous 10 years. The FGDs consisted of only women as they are primary bearers of reproductive, maternal, and child health outcomes, and best positioned to explain related challenges. In addition, the study employed Key Informant Interviews (KIIs) with 15 health workers who were selected based on their roles in maternal, reproductive, and child health service delivery at Kilwa District Council. These involved members from the council health management team and healthcare providers from dispensaries, health centers, and district hospitals. They were purposively selected to gain an in-depth understanding of how climate-related events impact maternal, reproductive, and child health service delivery. These insights complemented the lived experience shared by women in FGDs.

### Sampling

Seven wards were purposively selected for this study because they had recently experienced devastating floods and tropical cyclone Hidaya between March and May 2024. At least one focus group discussion (FGD) and one Key Informant interview (KII) were conducted in each selected ward. Local leaders and the district health management team assisted in identifying and recruiting study participants. A total of 102 participants (87 group discussants and 15 key informants) participated in this study. A purposive sampling technique was used to select individuals who could provide in-depth information on the topic under study [16].

### Data collection

A total of fifteen KIIs and ten FGDs, each consisting of 8 to 10 participants, were conducted. The KII and FGD semi-structured guide focused on exploring participants’ perceptions of climate change, the impacts of climate change on maternal, reproductive, and child health, and existing coping strategies to adapt to the changing climate. The KII and FGD semi-structured data collection tool guides were developed specifically for this study. The tool guides were prepared in English and translated into Kiswahili by the Principal Investigator (PI). All KIIs and FGDs were conducted in Swahili, as it is a *lingua franca* throughout Tanzania. All FGDs were moderated by the first author with the help of research assistants who were taking notes. On average, FGDs lasted about 1 h, while KIIs lasted about 35 min. Point of saturation was reached in the 7th FGD session and 10th KII; however, additional FGDs and KIIs were conducted to confirm the saturation.

### Data management, processing, and analysis

FGDs, and KIIs were recorded using a tape recorder (*Sony Voice Recorder, IPX470*), followed by transcription that was done verbatim. All audio recordings were transcribed and translated from Swahili to English. Data collected were cleaned to ensure they were correctly coded and labelled. Data analysis was done thematically, where open, axial, and selective coding were used. Open coding involved line-by-line transcript analysis to continuously compare the emerging concepts and categories. Conceptual and theoretical concepts that arose during data analysis were documented in memos. Data analysis was carried out continuously until theoretical saturation was attained. Finally, selective coding, which involves identifying and describing the major phenomenon or core categories within the data that best express the perspectives of research participants, was presented in a narrative report [12].

## Results

### Social Demographic Profile of Participants

A total of 102 participants took part in the study of whom 87 female respondents participated in the FGDs while the remaining 15 respondents (4 females and 11 males) participated in Key Informant Interviews. Ten (10) FGDs were held in total, with 8–10 participants per group. Women who participated in FGDs aged between 18 to 65 years with a median age of 35.5 years old. Most of them (74.7%) had primary-level education. The majority (90.8%) of the FGD respondents engaged in small-scale farming as their main economic activities (Table 1). A total of 15 KII were conducted. Most of the key informants were male (73.3%), aged predominantly between 31–35 years (33.3%), and held a diploma (60%). The majority were nurses (40%) or clinical officers (26.7%) with 1–5 years of work experience (53.3%). More details are shown in Table 2

**Table 1:** Socio-demographic profiles of FGD study respondents

Variable	Frequency (N)	Percentage (%)
Age		
18–27	22	25.3
28–37	31	35.6
38–47	27	31.0
48–57	7	8.0
Sex		
Male	0	0.0
Female	87	100
Marital Status		
Divorced	12	13.8
Married	63	72.4
Single	11	12.6
Widowed	1	1.1
Education level		
Diploma	3	3.4
Certificate	2	2.3
None	7	8.0
Secondary	10	11.5
Primary	65	74.7
Occupation		
Entrepreneur	3	3.4
Small-scale farmer	79	90.8
Health Assistant	1	1.1
Laboratory technician	1	1.1
Nurse	3	3.4

**Table 2:** Socio-demographic characteristics of key informants

Variable	Category	Frequency (N)	Percentage (%)
Age (Years)	26–30	4	26.7
	31–35	5	33.3
	36–40	4	26.7
	41–45	2	13.3
Sex	Male	11	73.3
	Female	4	26.7
Education level	Certificate	1	6.7
	Diploma	9	60.0
	Bachelor’s Degree and above	5	33.3
Professional role	Medical Doctor	1	6.66
	Environmental Health Officer	1	6.66
	Social Worker	1	6.66
	Nutrition Officer	1	6.66
	Nurse	6	40
	Clinical officer	4	26.7
	Medical Attendant	1	6.66
Working Experience	1–5 Years	8	53.3
	6–10 Years	4	26.7
	11–15 Years	3	20.0

### General perception about occurrence of climate change

When asked what they understood about climate change, most of the respondents described climate change as the changing of the seasons or shifting weather patterns. They also perceived it as a close and serious threat unfolding in their community having experienced devastating floods in May 2024. When further probed about the major changes in seasons, or weather events experienced in their community, the most mentioned were: extreme precipitation, unpredictable rainfall, and an increase in frequency and intensity of extreme weather events (EWEs) such as floods, drought, and increasing temperature. One of the participants explained;

*“There have been changes in the timing of the rainfalls. In the past, we used to experience normal rainfall seasons that were normal, but from as early as The 2020s, we experienced heavy rains that led to flooding and people had to relocate from their homes. Likewise, from 2023 to 2024, there was extreme rainfall which caused devastating floods we have never experienced before.” (FGD 03, Participant No.4, Kipindimbi Village*).

Another participant added;

*“According to my understanding, in the past years, the rain was normal and we knew when it would rain, and thus, we had time to prepare. Yet it was uncommon for rainfall to cause floods or any destruction. They were even predictable if it was raining in March, for sure you knew it would rain in March, but now if you expect rainy season in March, you will be surprised it is not raining……” (FGD 01, Participant No. 8, Nakingombe Village).*

Follow-up questions on rainfall patterns during the interview triggered more participants to share their experiences on how changing climate impacts their livelihoods particularly food and cash crop cultivation. One participant narrated that;

*“In the past, we used to prepare our farms in September expecting rain to start in December so that we can plant our seeds. But now in December, there is no rain as it used to be” (FGD 02, Participant No.10, Miguluwe Village).*

Another participant added on the occurrence of prolonged dry spells and floods;

*“Drought is also now more common compared to the past years. In 2023, it was too sunny and dry, pastoralists moved with their cattle into our area as grazing lands became scarce. In 2024, floods happened, and our crops were destroyed. The situation is bad” (FGD 04, Participant No.3, Mavuji Village).*

Respondents were also asked to explain about the causes of climate change. This question was felt to be more scientific and many people could not respond to it. However, one of the respondents mentioned about the factors that drive climate change;

*“In my understanding, two factors contribute to climate change: deforestation to grow sesame on a vast scale. Secondly, gas emissions from industries, also contribute to climate change” (FGD 01, Participant 8, Nakingombe Village).*

### Impacts of climate change on maternal, reproductive, and child health

Respondents reported that climate change negatively affects women’s and children’s health in many ways. Although some of the responses did not indicate a direct relationship between maternal, reproductive, and child health outcomes, our analysis has identified five sub-themes that emerged from the intensive discussions and interviews with the respondents. These include the increasing burden of climate-sensitive diseases, maternal and child malnutrition, mental health, limited access to health care services, and intensifying sexual and Gender-based Violence (SGBV). These are explained as follows;

#### Impacts of climate change on maternal and child nutrition

Respondents in this study reported that climate change has negatively affected maternal and child nutrition status in Kilwa District Council. They explained how unpredictable rainfall patterns and extreme weather events such as recurring droughts, floods, and tropical cyclone Hidaya have disrupted agricultural productivity, resulting in food insecurity. Several participants pointed out that the recent devastating flood in low-lying areas destroyed their farmlands and left many households with food insecurity. One of the participants said;

*“Regarding food availability, it is now a challenge because all farming fields have been flooded, and we cannot cultivate. In this situation, food availability has significantly reduced as our farms in low-lying areas have been flooded with water” (FGD 02, Participant No.1, Kipindimbi Village).*

To cope with this situation, many households resort to reducing the quantity and number of meals taken per day. When asked about the most common type of food consumed by pregnant and lactating mothers and children, starchy foods such as stiff porridge (ugali) were highly mentioned by the respondents. One of the respondents said;

“*You may find a woman making cassava porridge in the morning, in the afternoon she has nothing to eat, and in the evening, she cooks ugali (stiff porridge) to eat with her children. She still has to leave some for her children to eat before going to school the next day. Only God understands the challenges they face.” (FGD 02, Participant No. 4, Miguluwe Village).*

Furthermore, participants noted that climate-related extreme events, such as droughts and floods, have reduced access to and consumption of iron-rich foods, especially green vegetables, which are the primary local source of dietary iron in their community. Unpredictable rainfall, drought, and flooding were reported to make home or small-scale vegetable farming increasingly difficult. Healthcare providers (key informants) perceived anemia as the most common health issue among pregnant women and children under five years of age in their communities and attributed it to decreased access and consumption of green vegetables. One of the key informants said;

*“As a result of that challenge, many pregnant women and many children suffer from anemia. If you look at the top ten diseases, anemia ranks first because there are no green vegetables”(FGD 09, Participant No.5, Tingi Village).*

Some participants perceived a connection between climate-induced food insecurity and adverse birth outcomes such as low birth weight. Participants explained that during periods of flooding or prolonged dry seasons, pregnant women often have less access to nutritious foods, which they believed negatively affects the health and growth of the foetus. One participant said:

*“… giving birth to underweight children is increasing in our communities, and many cases occur during flooding or summer; therefore, we believe reduced access to sufficient nutritious food contributes to malnutrition.” (FGD 04, Participant No.5, Mavuji Village).*

#### Increased prevalence of climate-sensitive diseases

Discussants and key informants expressed their concerns about increased cases of some diseases following the heavy rainfall season and rising temperatures. The most common climate-sensitive diseases mentioned were malaria, diarrhea, heat rash, urinary tract infections, and cholera. It was reported that many pregnant women and children suffer more from Malaria in the district. This was linked to climatic conditions such as rainfall and temperature that create a suitable environment for the growth and survival of disease-carrying vectors such as mosquitoes.

Participants reported noticeable changes in seasonal weather patterns in recent years. They reported in the past, between May and August, it used to be cold season, but it has now become warmer. This observation was perceived to be associated with the increase in the mosquito population, which in turn contributes to an increase in malaria transmission throughout the year. One of the participants mentioned;

*“In the past, the cold season started in May and ended in August. But now it is very hot and the mosquito population is relatively high in both seasons” (FGD 03, Participant No. 6, Kipindimbi Village).*

Additionally, participants have observed a rising number of malaria cases, particulary during rainy seasons, and in the aftermath of the recent devastating floods. Healthcare providers perceived that the observed increase in malaria cases after the recent floods in April 2024 was due to a lack of utilization of mosquito bed nets among the displaced population who were residing in shelters such as schools. Pregnant women and children were reported as the most at-risk population groups. Contracting malaria during pregnancy was reported to result in serious complications among pregnant women, such as increased risks of premature birth. Notable comments from the respondents illustrate this perceived climate-related health risk:

*“[…….]Some of the children were sleeping in schools. They were suffering most from Malaria because they did not have mosquito nets and the mosquito population was high” (FGD 09, Participant No.5, Tingi Village).*

*“During that season, malaria prevalence was high, and some of the pregnant women who contracted malaria gave birth prematurely” (FGD 09, Participant No.5, Tungi Village).*

Respondents also mentioned the increasing prevalence of water-borne diseases following the flood season. It was reported that the main drinking water sources were contaminated with floodwater, and this was associated with the outbreak of diarrhoea disease in the district. One of the key informants narrated that;

*“… People fetch water that is already contaminated by the floods. For example, this year there has been an outbreak of diarrhoea due to heavy floods, something we had almost forgotten about here in Kilwa. But it has happened this year, and it continues to persist because people are consuming drinking water from the ponds. (KII 02, In Charge of Health Facility, Kinyonga District Hospital).*

Another participant added that;

*“…. I would like to talk about my experience during flooding. Most of the children were suffering from diarrhea. This diarrhea may be due to children collecting water from rivers and drinking” (FGD 04, Participant No.1, Mavuji Village).*

#### Limited access to healthcare services

Respondents reported that floods and tropical cyclone Hiday in 2024 introduced challenges in accessing healthcare services. Most of them confirmed crucial infrastructures such as roads, and bridges were damaged making it hard to reach the healthcare facilities. As a result of this seasonal barrier, participants revealed pregnant women and mothers faced challenges in attending antenatal and post-natal clinics. Some respondents shared the lived experience of a few pregnant women in their communities delivering without assistance from skilled attendants as a result of flooding. One respondent had this to say:

*“Pregnant women face health risks. For example, sometimes roads become impassable therefore some of the pregnant women deliver at home, and some of them may even lose their babies…” (FGD 09, Participant No.4, Tingi Village).*

Another healthcare worker shared his experience of assisting a pregnant woman to deliver safely at home as she was unable to reach the facility in time due to flooding. The participant said;

*“…. many places in the village were flooded, I remember I attended three cases, where I had to assist with home deliveries because they couldn’t make it to the healthcare facility. In other cases, you may find she has already given birth, so I would just give her support. There were several cases like these during flooding. There was a period when even healthcare workers could not reach the facility, it was very challenging because of floods (KII 15, Health worker, Somanga Health Center).*

*Other participants also stated;*

*“For a pregnant woman, when there are heavy rains and the river overflows, it becomes difficult for her to attend clinic. It also happens children fall sick but they cannot access timely healthcare services.” (FGD 01, Participant No. 6, Nakingombe Village).*

Failure to attend clinics was reported to have serious impacts on maternal and child health. Respondents mentioned this can result in poor health outcomes and other childbirth complications. One of the key informants mentioned;

*“When a mother comes to the clinic, it is not only for check-ups but also for health education. We provide them with health education. For example, for pregnant mothers, we test their blood levels every week, but also through those clinics, they are given drugs and supplements, such as deworming drugs, antimalarial drugs, and iron supplements to increase the amount of blood. So, if the mother does not attend the clinics, it becomes a challenge. many pregnant women may lose their pregnancies because they do not attend the clinic.” (KII 15, Health worker, Somanga Health Center).*

#### Climate change and fertility intentions

As a result of the increased vulnerability of pregnant women to the changing climate, women fear carrying a pregnancy because of the worsening climate crisis, especially recurring floods. Many of the participants described not wanting to have more children in the wake of climate-related disasters, as they found it challenging to access essential reproductive health services such as safe childbirth and meet basic needs for children, such as feeding and taking them to school. One of the participants mentioned that family planning is the best option they rely on to avoid the associated challenges of having a pregnancy in the wake of climate disasters. She said;

*“Yes, we are afraid of getting pregnant because of the road conditions during the rainy season. Therefore, you go to the health facility and get an implant that will last for five years, others may prefer pills or injections. If the roads are impassable there is no way you may choose to take the risks to get pregnant” ((FGD 02, Participant No.4, Miguluwe Village).*

Existing financial hardship associated with the economic losses from low yields of food and cash crops was also linked to changes in fertility intentions. The same participant added that;

*“You are living with your husband, and you don’t have five thousand shillings. What will happen when you get pregnant? Nowadays, being pregnant is expensive, in just the first week you must incur some costs until you reach nine months….” (FGD 02, Participant No.4, Miguluwe Village)*

### Intensifying sexual and gender-based violence

Women are disproportionately impacted by climate shocks due to patriarchal gender norms that not only position them as primary providers of food and care but also assign them the responsibility of securing household water. During periods of prolonged dry spells and erratic rainfalls, women and girls are forced to travel longer distances to search for water. This extended journey to water collection points exposes women, especially young girls, to heightened risks of experiencing sexual harassment. Without accompaniment by adults, the girls encounter the risks of being raped by men. One of the participants articulated this concern by pointing out that;

*“Sometimes the mother may be overwhelmed with other household tasks and thus may request help from her young girl. When she goes there, she may find a lot of containers lined up, and other people may not consider that she will be late going back home. These water sources are located in distant areas, and often, children are sent to fetch water there. Recently, pastoralists have moved into our areas, and their minds are different from ours. When they see young girls, they take advantage and rape them” (FGD 02, Participant No 1, Miguluwe village).*

Additionally, as a result of the climate crisis, women's burden in traditional roles increases. They spent more time undertaking the household chores, which often led to physical exhaustion. Domestic violence sometimes arises when women are perceived as unwilling or unable to engage in sexual relations with their partners due to fatigue. This fuels frustration among their partners, which often results in domestic violence. One of the participants said;

*“When women return from the farm, they go to search for firewood and vegetables therefore, they do not get adequate rest. Even when the husband wants sex, he will be frustrated as he thinks she is denying him sex, leading to verbal abuse and domestic violence.” (FGD 02, Participant No.10, Miguluwe Village).*

Additionally, another respondent added that women might be questioned by their partners about being tired from such tasks and being beaten by their husbands, she said;

*“Ooh! You are tired?… but you have fetched water, why are you tired to do this, you are beaten and experience all sufferings” (FGD O2, Participant No.4, Miguluwe Village).*

Also, participants reported that climate-induced economic instability increases the risks of sexual and gender-based violence in contexts where women are dependent on men’s role as breadwinners in households. Climate shocks reduce households'incomes through compromised means of livelihoods such as agriculture, which undermines men’s role to provide for their families and also exacerbates household poverty. In some cases, this situation compels young children, particulary young girls, to engage in high-risk coping practices such as transactional sex to meet their basic needs. One of the respondents mentioned;

*“…Also, many women were experiencing abuse from their husbands due to a lack of resources in the family. When parents separate, and the mother has no means of gaining income to feed her family, the children are forced to find means of living on their own; sometimes they are being sexually exploited to get money. For the young girls, they may be raped, so it becomes a bit of a challenge.” (FGD 09, Participant No.7, Tingi Village).*

One of the key informants said that;

*“Aaaah… those issues are also common here. When you look at registry, many young girls below 18 years of age are pregnant and when you investigate, you find poverty contributes much to this; therefore if they are offered something, they are easily influenced. Such cases are many in our community, and if you look at our register, you will find the age of first-time mothers is around 15, 16, 17, or 18. They are still young girls” (KII 15, Health worker, Somanga Health Center).*

### Existing adaptation measures to address the impacts of climate change

The extreme weather events impose seasonal barriers to access health facilities, especially for pregnant women who live far away or across difficult terrain from healthcare facilities. This is coupled with limited transportation options. One of the key informants mentioned in the District Hospital, a Maternity Waiting Home (MWH) has been constructed where pregnant women stay before giving birth. This helps many pregnant women access essential healthcare services including timely safe deliveries at all times. One of the key informants said;

*“……in our district hospital, we have a designated building called Mama Ngojea. This is a building that accommodates pregnant women who come from remote areas, therefore they come here early so that they may not face any barriers when it is time to give birth” (KII01, DMO, Kilwa District Council).*

Additionally, psychosocial support is given to climate-related disasters such as flood victims. The key informant mentioned;

*“There are organizations that provided psychosocial support conducted during village meetings for flood victims who lost their properties to build their resilience ……” (KII 01, DMO, Kilwa District Council).*

There is also relocation of the community members from flood-prone areas to safe grounds. One of the health workers said;

*“…Njinjo is one of the villages that was completely wiped out two or three years ago. There is a river called ‘Matandu’ which severely flooded the village. The rains were pouring in nearby areas like Morogoro, but the flood water reached Njinjo and submerged the whole village. Due to this, they decided to relocate the village to another safe area…..” (KII 02, In Charge of Health Facility, Kinyonga District Hospital).*

## Discussion

This study aimed to explore community and healthcare workers'perspectives on the impacts of climate change and related extreme weather events on reproductive, maternal, and child health outcomes in Kilwa District Council. The findings from this study contribute to the ongoing dialogue concerning the nexus of climate change and maternal, reproductive, and child health, specifically through the perspective of community and health workers in Tanzania. Our results revealed that most respondents demonstrated adequate knowledge regarding climate change by describing rising temperatures, unpredictable rainfalls, increased frequency of floods, and droughts as key evidence of climate change over time, echoing findings from Eastern Kenya. Participants also acknowledged anthropogenic activities such as industrial pollution and deforestation as the main causes of climate change, consistent with studies in Kenya and the Mediterranean Islands. Our findings demonstrate that awareness may increase following immediate experience of climate-related events or disasters. This post-disaster scenario offers an opportunity to further evaluate or study how high-risk perceptions may influence individuals'and communities'adoption of climate-resilient health behaviours over time and the development of effective, context-specific adaptation strategies.

A significant contribution of our study was the exploration of reproductive, maternal, and child health outcomes and healthcare service delivery in the context of extreme weather events such as floods in Tanzania. This study shows that floods temporarily hamper accessibility to health facilities and delivery of maternal and child health services in Kilwa District by damaging transportation networks and increasing travel costs, preventing women from reaching facilities for safe deliveries, antenatal care, and essential health interventions such as deworming and vaccinations. These disruptions increase the risks of preventable maternal and neonatal deaths and echo findings reported in other low- and middle-income countries [10, 24, 27, 31, 32]. The results underscore the critical need to design and empirically evaluate the most effective health service delivery models that can ensure health service continuity during climate-related emergencies. In addition, the findings point to the critical urgency of integrating these services in emergency preparedness plans and climate change policy frameworks as deliberate efforts to ensure that health service delivery becomes resilient to the increasingly climate-related disasters.

Our study also highlights reported increases in cases of climate-sensitive diseases, such as malaria and diarrhea, which were perceived as serious public health concerns after a flooding event in the Kilwa district. The stagnant flood water was reported to provide breeding sites for mosquitoes and habitats for other waterborne pathogens. Pregnant women and children under five years old were identified as the most vulnerable groups. Our findings reflect results reported in several studies conducted in flood-prone areas like Pakistan (M. A. [2, 7]). These findings point out that climate-related extreme weather events have the potential to exacerbate existing public health challenges in resource-constrained settings such as developing countries, which necessitates the need for strengthening health system preparedness through improving early warning systems for climate-sensitive diseases and targeted interventions to reduce the burden of diseases when they arise during climate-related emergencies. Further, more epidemiological and physiological studies are needed in Tanzania to establish a causal association between negative health outcomes perceived by the respondents, such as malaria, anemia, diarrhoea, heat rashes, and the ongoing climate crisis.

Moreover, the study reveals that many households face food insecurity due to the destruction of farm fields and loss of food crops, following devastating floods and tropical cyclone Hidaya that struck the district between April and May 2024. The climate-related shocks destabilize the economy, which leads to secondary impacts on household food security. Reduced food availability was perceived to increase the risks of malnutrition among pregnant women and children under five years of age. Similar results from Bangladesh and a global systematic review reveal that climate-induced food insecurity is among the drivers of maternal and child malnutrition in flood-prone areas [19, 24]. The findings highlight the need to consider the specific nutritional requirements of pregnant women and children in emergency response and relief programmes, and implement long-term strategies for strengthening the resilience of local food systems.

Our study findings reveal that women and girls are disproportionately impacted by climate change than their male counterparts, primarily due to existing patriarchal gender norms in society, which assign them primary responsibilities such as fetching water, preparing food, while limiting their access to resources, and participation in decision-making. These socially constructed roles increase the burden on women, especially when they are forced to travel longer distances searching for water, which becomes scarce as a result of prolonged dry spells, which put them at greater risk of exposure to sexual and gender-based violence. Our findings resonate with global evidence showing that climate crises are associated with increased cases of sexual and gender-based violence, including domestic violence and child marriage, worldwide [4, 6, 8, 9]. This highlights that climate change has taken a gender dimension as a result of differential vulnerability between men and women. This implies that effective climate adaptation measures should take into account the gendered impacts of climate change, including strategies to challenge patriarchal norms that restrict women's access to resources and participation in decision-making. Additionally, this calls for the formulation of gender-responsive climate policy frameworks that recognize, prioritize, and address the unique vulnerabilities and needs of women.

While the study provides a unique exploration of the community and healthcare workers’ perspectives on the impacts of climate change and related extreme weather events in one of the most climate-vulnerable districts, it has several limitations. Foremost, it was conducted in only one flood-prone district and exclusively relied on qualitative perceptions of the respondents, and lacked statistical grounding of quantitative analyses; therefore, the generated findings may not be generalizable across Tanzania. The focus on rural coastal districts, while being critical, may not fully represent the conditions in other settings, including urban areas, and other regions, taking into account that climate variability and its related impacts are context-specific. Moreover, the findings may be subject to inherent biases associated with individuals and professional experience, which may potentially influence the study’s outcomes and interpretations.

## Conclusion

Our study respondents demonstrated clear perceptions regarding changes in climate over the last ten years, as evidenced by rising temperatures, unpredictable rainfalls, warmer winters, and increased frequency and intensity of extreme weather events such as floods and tropical cyclones. Study participants also perceived the impacts of climate change and related extreme weather events on human health and the health system. They described how maternal and child health were directly and indirectly affected by rising temperatures, unpredictable rainfall, and floods. Limited access to health facilities, compromised delivery of maternal and child health services, changes in fertility intentions, surging cases of climate-sensitive diseases such as malaria, diarrhoea in the post-flood scenario were specifically mentioned. Most of the community and healthcare workers'perceptions on the impacts of climate change on reproductive, maternal, and child health were consistent with the existing epidemiological and physiological evidence. Being the first study of its kind in Tanzania, particularly in a district that experiences extreme weather events (recurring floods and tropical cyclones), these findings contribute to a much-overlooked area. The findings point to the critical urgency of prioritizing reproductive, maternal, and child health in climate policies, including the Health National Adaptation Plan (HNAP), the Nationally Determined Contributions (NDCs), to ensure delivery of the RMNCAH services is resilient to the changing climate. This also calls for full integration of climate change considerations in existing health sector policies, strategies, plans, programmes, and interventions as deliberate efforts to strengthen healthcare system resilience.

## Supplementary Information

Supplementary Material 1.

Supplementary Material 2.

Supplementary Material 3.

## Data Availability

The data analyzed during the current study will be available from the co-corresponding author upon reasonable request. Interested parties should contact Dr. Hussein Mohamed at hmohameds1@gmail.com.

## Abbreviations

EWEs: Extreme Weather Events
FGD: Focus Group Discussion
KII: Key Informant Interview
MUHAS: Muhimbili University of Health and Allied Health Sciences
MWH: Maternity Waiting Home
PTSD: Post-traumatic stress disorder
SGBV: Sexual and Gender-based Violence
SRH: Sexual and Reproductive Health
RMNCAH: Reproductive Maternal, Neonatal, Child, and Adolescent Health
NDC: Nationally Determined Contributions
H-NAP: Health National Adaptation Plan
