# Supplementary material for: Community and health workers’ perspective on impacts of climate change on reproductive, maternal, and child health outcomes in Kilwa district council, Tanzania: a qualitative study
Source: BMC Public Health. 2025 Sep 30;25:3185. doi: 10.1186/s12889-025-24343-2 (PMC12487242; doi:10.1186/s12889-025-24343-2)
Supplement: Supplementary file 1 — Supplementary Material 1. [file 12889_2025_24343_MOESM1_ESM.pdf]

Semi-structured focus group discussion data collection guide

Research Title: “Climate Change and Maternal, Reproductive and Child Health Outcomes in Tanzania: Evidence from Qualitative Study in Kilwa District, Lindi Region”

GUIDING QUESTIONS:

1. What do you understand by the term climate change?
   Probe:
   a) Do you perceive climate change as a serious threat in your community? Why?
   b) What changes in weather/climate have you noticed happening in Kilwa District Council in recent years? (*Recurring floods, droughts, tropical cyclones, rise in temperature, sea level rise, prolonged dry spells, unpredictable rainfall, etc.)*
   c) Which of those weather/climate changes occur more frequently or more severely compared with the past years?
   d) Who is more affected in your community and why? Probe more on;
      i. How are pregnant women affected change?
      ii. How are children affected by climate change?
      iii. How are other vulnerable groups affected by climate change?
   e) Where do you often get information about climate change in your community?

2. How does climate variability such as *floods, droughts, heatwaves*, etc., affect maternal, reproductive, and child health in your community?
   Probe on the following;
   a) How have climate-sensitive diseases such as *malaria, diarrhoea, cholera*, etc. changed in your community?
      Probe more on;
      i. What do you think are the reasons for such changes in the prevalence of climate-sensitive disease in your area?
      ii. What are the most common diseases affecting pregnant women?
      iii. What are the most common diseases affecting children
      iv. What disease outbreaks have ever occurred after events such as floods or heavy precipitation in your community?
   b) How does climate variability impact the availability of nutritious food in your area?
      Probe;
      i. How does climate variability impact food production in your areas?
      ii. What are the impacts of reduced food production on household access and consumption of nutritious food?
      iii. How does food insecurity affect pregnant women and children in your community?

iv. What are common types of food consumed by the majority of households in your community?
v. Are there specific nutritious foods available for pregnant women or children? What are those?

c) How does climate variability affect access to healthcare services in your area?
   i. How do extreme weather events such as heavy precipitation and floods affect transportation to healthcare facilities in your area?
   ii. Have you or anyone you know ever experienced failure to access healthcare facilities due to extreme weather events? Please describe your experience.
   iii. Have you ever experienced or heard from your neighbors about pregnant women facing challenges in reaching health facilities due to floods or any other extreme weather condition?
   iv. Can you describe experiences on home deliveries or unattended births that occurred in your community because the pregnant woman was unable to reach the healthcare facility due to floods or any other extreme weather events?
   v. Does climate variability influence people's fertility intentions? How?
d) How does the climate crisis influence sexual and gender-based violence in your community?
   i. How does resource scarcity, such as water, food has influence or lead to domestic violence in your community?
   ii. How does the climate crisis influence sexual violence in your community?
   iii. Are there any reported cases of child marriage that are associated with climate variability?
   iv. How does climate-induced food insecurity and poverty influence abuse of women and girls in your community?

3. What are the existing adaptation measures/coping strategies that you implement in response to climate change? In other words, what do you do in cases of the following extreme weather events?
   a) Heatwaves,
   b) Extreme rainfall/precipitation
   c) Drought e.g. uses big water reservoirs, use drought resistant seeds, irrigation, store food in case drought comes etc.
   d) Floods
   e) Unpredicted rainfall

4. Are the above adaptation measures effective in addressing climate-related challenges? Why?
   Probe: Any challenge faced in implementation of these adaptation measures.

5. Is there anything you would like to add that has not been covered in this discussion?
