# Supplementary material for: Community and health workers’ perspective on impacts of climate change on reproductive, maternal, and child health outcomes in Kilwa district council, Tanzania: a qualitative study
Source: BMC Public Health. 2025 Sep 30;25:3185. doi: 10.1186/s12889-025-24343-2 (PMC12487242; doi:10.1186/s12889-025-24343-2)
Supplement: Supplementary file 2 — Supplementary Material 2. [file 12889_2025_24343_MOESM2_ESM.pdf]

Semi-structured Key Informant data collection guide

Research Title: "Climate Change and Maternal, Reproductive and Child Health Outcomes in Tanzania: Evidence from Qualitative Study in Kilwa District, Lindi Region"

GUIDING QUESTIONS:

1. What do you understand by the term climate change?
   Probe:
   a) Do you perceive climate change as a serious threat to public health? Why?
   b) What changes in weather/climate have you noticed happening in Kilwa District Council in recent years? (*Recurring floods, droughts, tropical cyclones, rise in temperature, sea level rise, prolonged dry spells, unpredictable rainfall, etc.)*
   c) Which of those weather/climate changes occur more frequently or more severely compared with the past years?
   d) Who are the most vulnerable groups to the climate-related health risks in your area? And why?

2. How does climate variability such as *floods, droughts, heatwaves*, etc., affect maternal, reproductive, and child health in your community?
   Probe on the following;
   a) What are the common climate-sensitive diseases that affect pregnant women and children in your area?
   b) How does climate-induced food insecurity impact maternal and child nutrition status in your area?
   c) How does climate variability affect access and/delivery of essential healthcare services in your area?
      i. What are the most likely impacts of compromised healthcare services delivery?
      ii. Please describe any event/scenario on the consequences of compromised healthcare service delivery during climate-related disasters.

3. What are the existing adaptation measures/coping strategies that you implement in response to climate change?
   a) How does your healthcare facility ensure minimal disruption in the provision of healthcare services during climate-related disasters such as floods?
   b) Please describe your experience with healthcare service delivery during the latest flooding events in the Kilwa district.
   c) Are the adaptation measures effective in addressing climate-related health risks? Why?

4. Is there anything you would like to add that has not been covered in this discussion?
