# Supplementary figures and images for: Community and health workers’ perspective on impacts of climate change on reproductive, maternal, and child health outcomes in Kilwa district council, Tanzania: a qualitative study
Source: BMC Public Health. 2025 Sep 30;25:3185. doi: 10.1186/s12889-025-24343-2 (PMC12487242; doi:10.1186/s12889-025-24343-2)

**Figure 1: Map of United Republic of Tanzania showing study area (Kilwa District Council)**

*
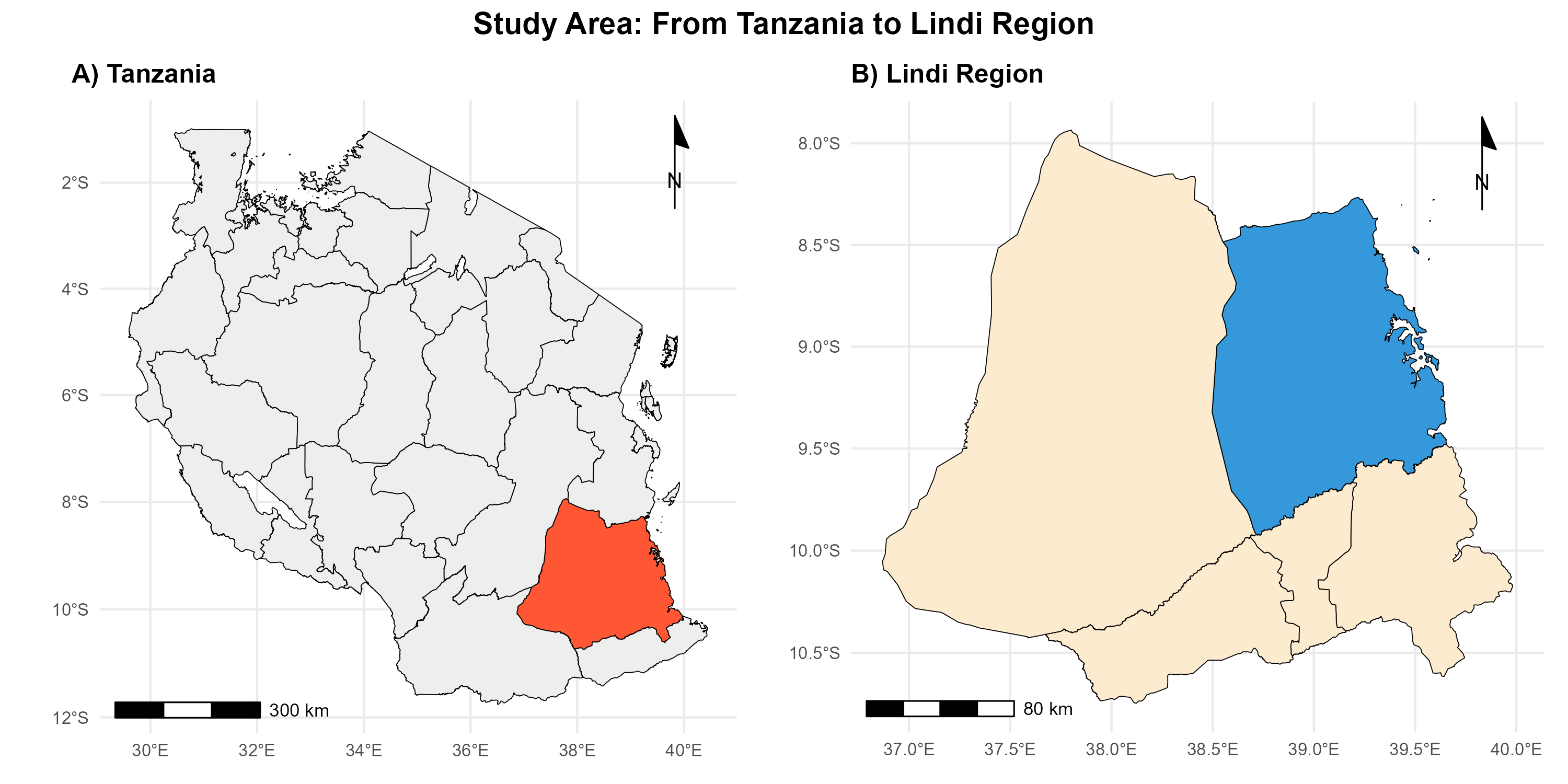
*

*
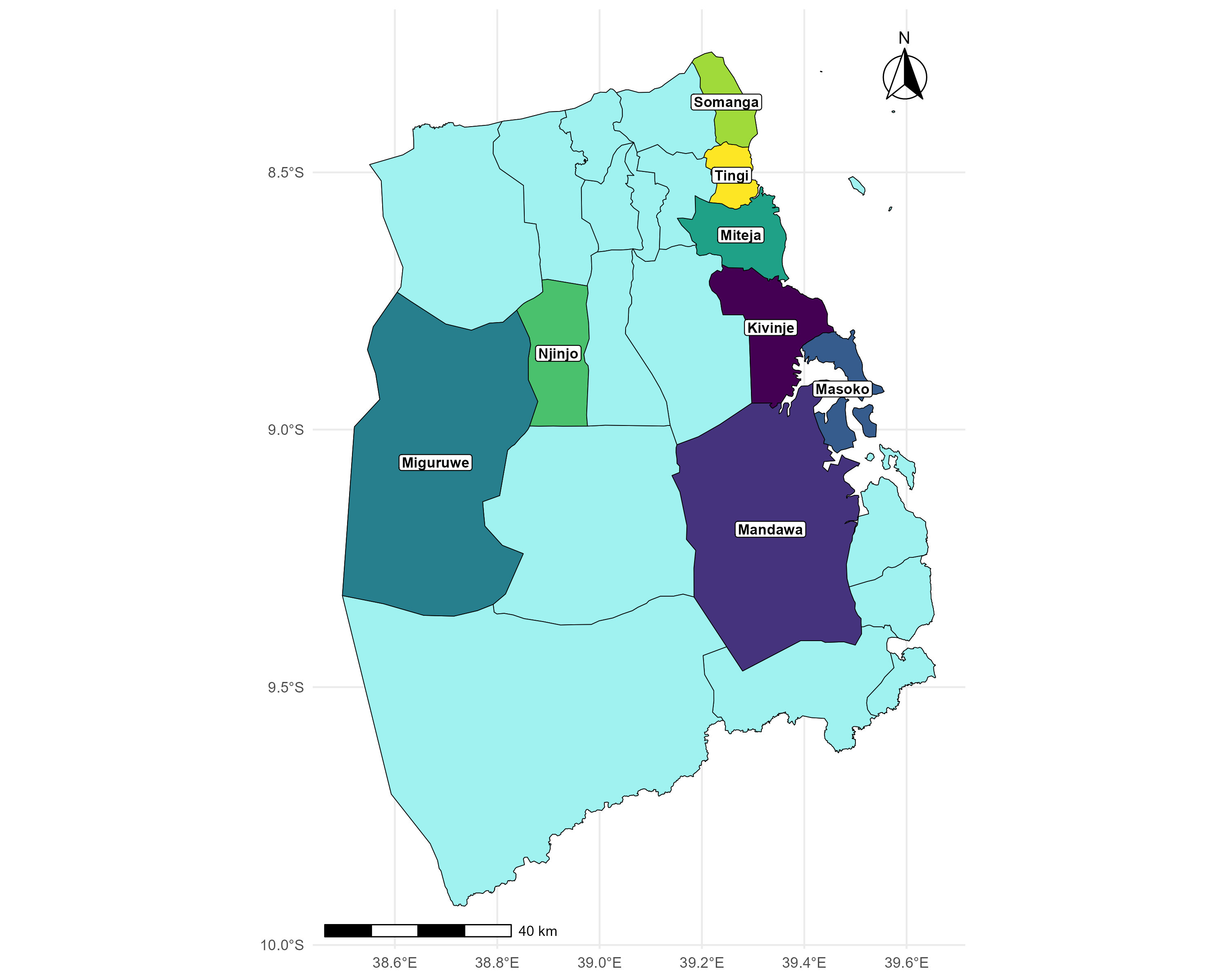
*

Supplement: Supplementary file 3 — Supplementary Material 3. [file 12889_2025_24343_MOESM3_ESM.docx]
